# Solar active region magnetogram image dataset for studies of space weather

**DOI:** 10.1038/s41597-023-02628-8

**Published:** 2023-11-24

**Authors:** Laura E. Boucheron, Ty Vincent, Jeremy A. Grajeda, Ellery Wuest

**Affiliations:** Klipsch School of Electrical and Computer Engineering, New Mexico State University, Las Cruces, NM 88003 USA

**Keywords:** Solar physics, Electrical and electronic engineering, Scientific data

## Abstract

In this dataset we provide a comprehensive collection of line-of-sight (LOS) solar photospheric magnetograms (images quantifying the strength of the photospheric magnetic field) from the National Aeronautics and Space Administration’s (NASA’s) Solar Dynamics Observatory (SDO). The dataset incorporates data from three sources and provides SDO Helioseismic and Magnetic Imager (HMI) magnetograms of solar active regions (regions of large magnetic flux, generally the source of eruptive events) as well as labels of corresponding flaring activity. This dataset will be useful for image analysis or solar physics research related to magnetic structure, its evolution over time, and its relation to solar flares. The dataset will be of interest to those researchers investigating automated solar flare prediction methods, including supervised and unsupervised machine learning (classical and deep), binary and multi-class classification, and regression. This dataset is a minimally processed, user configurable dataset of consistently sized images of solar active regions that can serve as a comprehensive image dataset of LOS photospheric magnetograms for solar flare prediction research.

## Background & Summary

In this dataset, we provide a comprehensive collection of line of sight (LOS) solar photospheric magnetograms (images quantifying the strength of the photospheric magnetic field) from the National Aeronautics and Space Administration’s (NASA’s) Solar Dynamics Observatory (SDO). SDO was launched on 11 February 2010 as the first mission of the Living With a Star (LWS) program which seeks to understand solar variability and the effects of space weather at Earth and throughout the Solar System^1^. Specific goals of SDO in line with this dataset are to better understand the magnetic structure of the Sun and understand and predict how that magnetic structure initiates space weather events such as flares^1^. Three experiments are included on SDO: the Atmospheric Imaging Assembly (AIA)^2^, the EUV Variability Experiment (EVE)^3^, and the Helioseismic and Magnetic Imager (HMI)^4^. In this paper, we focus on LOS magnetogram images from HMI.

The dataset presented in this paper provides a comprehensive set of HMI magnetograms of solar active regions (regions of large magnetic flux, generally the source of eruptive events) as well as labels of corresponding flaring activity. This dataset will be useful for research on solar image analysis, particularly that related to magnetic structure, its evolution over time, and its relation to solar flares (a sudden and large emission of radiation). It is expected that the user community for this dataset will be researchers investigating automated solar flare prediction methods, including supervised and unsupervised machine learning (classical and deep), binary and multi-class classification, and regression. While SDO provides an incredibly rich dataset that can be an excellent source for image processing and machine learning researchers, there are several characteristics of the data that motivated our creation of this specific dataset. First, and overarching, was the desire to provide a minimally processed, user configurable dataset that can serve as a comprehensive image dataset for solar flare prediction research utilizing photospheric magnetograms, alleviating the need to download and curate a custom dataset. Second was the desire to focus analysis on solely active regions and to reduce the amount of time needed to interact with existing interfaces to download such data. Third was the desire that images of those active regions be consistently sized images rather than varying across active regions and/or across time, an important characteristic for standard deep learning architectures. Fourth was the necessity of integrating a separate dataset of flare strengths in order to provide labels related to flare activity for each image in the dataset.

Other studies in flare prediction using magnetograms commonly use the Space-Weather HMI Active Region Patches (SHARPs)^5^. SHARPs provide cutouts (“patches”) around HMI Active Region Patches (HARPs) which are concentrations of high magnetic flux which do not necessarily correspond to National Oceanic and Atmospheric Administration (NOAA) active region (AR) numbers. The patches include the photospheric vector and LOS magnetic field, Doppler velocity, and continuum intensity. Additionally, summary parameters are extracted for each SHARP, including features found to be associated with increased flaring behavior. Many studies of flare prediction use SHARPs, including use of the parameters (or subsets), e.g.^6–16^, proposing additional parameters extracted from the patches, e.g.^6,16–19^, and/or using the patches themselves, e.g.^9,12–15,20–25^. We choose not to use the SHARP dataset as the patches vary in size between HARPs, making the data incompatible with common deep learning architectures like convolutional neural networks (CNNs) that assume a fixed-size input, e.g., 224 × 224 pixels for AlexNet^26^, VGG^27^, and ResNet^28^ and 299 × 299 pixels for Inception^29^. Datasets of full-disk HMI LOS magnetograms were used in^30–32^ and parameters related to full-disk measurements were used in^33^, whereas we focus on the analysis of individual ARs. We choose LOS magnetograms over vector magnetograms to minimize dataset size. Datasets of HMI LOS magnetograms of ARs were used to extract features for use in classification in^34^, and additional features from AIA images were added in^35,36^; these datasets focus on features of ARs rather than providing a dataset of the underlying magnetograms as we do. A dataset of HMI LOS AR magnetograms was used in^37^, and of HMI LOS AR magnetograms and intensitygrams in^38^, but those datasets do not appear to be publicly available. Publicly available datasets include full-disk AIA images^39^ and 3D extrapolations of magnetic fields^40^, but do not make available LOS AR magnetograms as we do.

## Methods

### Dataset overview

As described above, there were four overarching characteristics that motivated our creation of this specific dataset: (1) a minimally processed, user configurable dataset that can serve as a comprehensive image dataset for researchers investigating the use of LOS magnetograms for flare prediction, (2) a focus on ARs with a reduction in time needed to interact with existing interfaces that can provide cutouts of ARs, (3) a dataset with consistently sized images for compatibility with common deep learning architectures, and (4) an integration of flare strengths as labels related to flare activity for the dataset. In this dataset, we address the aforementioned characteristics as follows. First, we provide a comprehensive set of magnetogram images from all NOAA ARs from May 2010 through December 2018. Along with this set of images, we provide a means to configure basic parameters of the dataset, including the strength of flares to consider, the time window over which to consider flare prediction, the latitudes and longitudes of active regions to include, and whether to include images with Not-a-Number (NaN) pixel values. Second, we integrate two sources of data in order to retrieve data only associated with ARs and provide a means to automate the download of those AR magnetogram images. Third, we provide consistently sized (600 × 600 pixel) images, which can be an important assumption in batch processing of images, particularly for some common deep learning methods, e.g., CNNs^26–29^. Fourth, we integrate a third source of data in order to provide labels related to flaring activity.

**Fig. 1:**
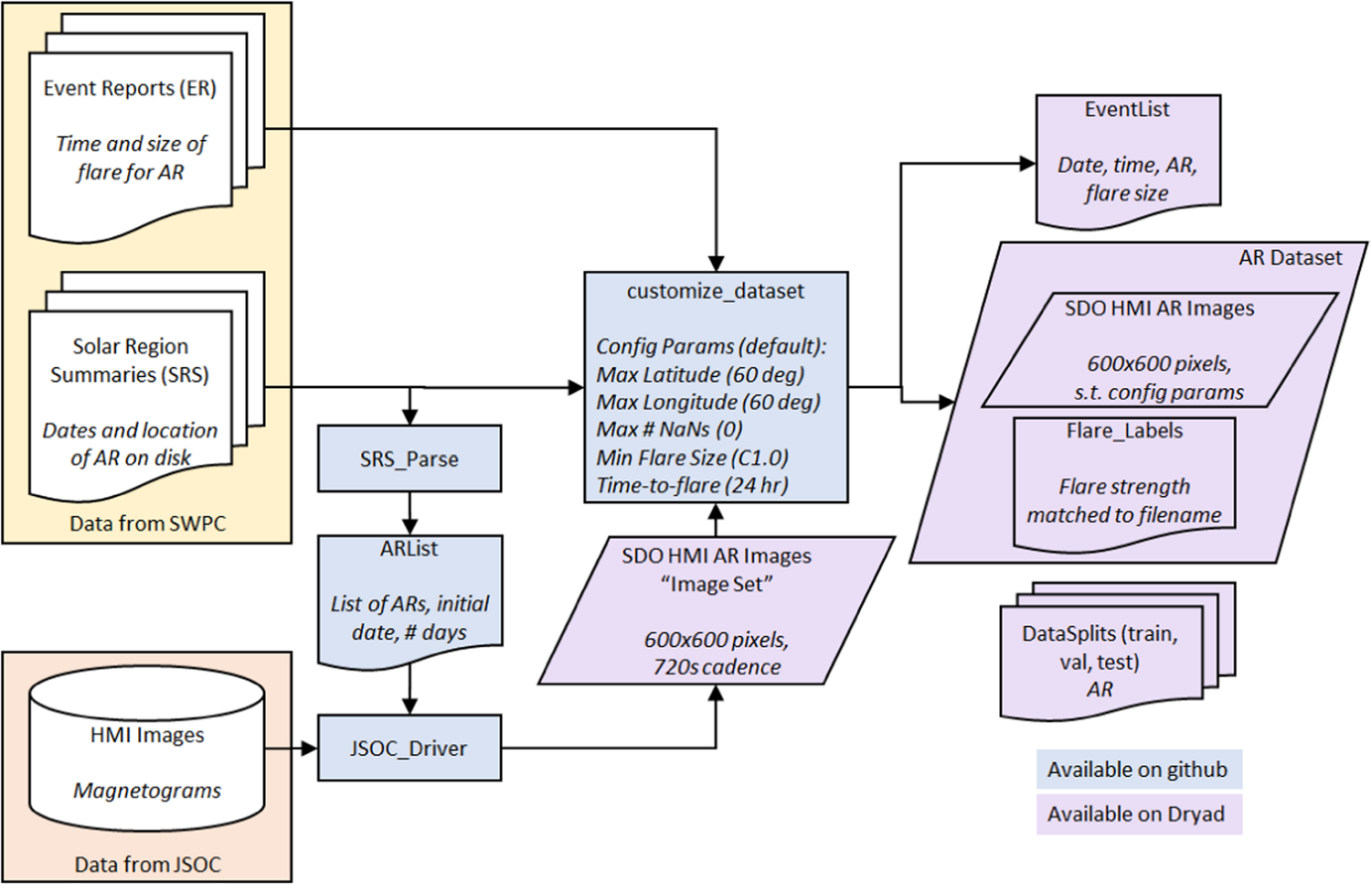
Flowchart of dataset creation. Space Weather Prediction Center (SWPC) Solar Region Summaries (SRS) are used to determine the dates for which a National Oceanic and Atmospheric Administration (NOAA) Active Region (AR) is visible on disk. Solar Dynamics Observatory (SDO) Helioseismic and Magnetic Imager (HMI) magnetogram images of ARs are downloaded via the Joint Science Operations Center (JSOC) web interface. SWPC Event Reports (ER) are used to specify the time and strength of solar flares associated with a given NOAA AR.

This dataset incorporates data from three main sources. First, in order to focus the image collection on ARs, we used the NOAA Space Weather Prediction Center (SWPC) Solar Region Summaries (SRS) (ftp://ftp.swpc.noaa.gov/pub/warehouse/) and parsed those text data to extract the date an AR appeared on disk and the number of days it was visible on disk. Additionally, the SRS provide latitude and longitude of ARs which we use to postprocess the dataset. Second, we download magnetogram images from SDO/HMI using the Joint Science Operations Center (JSOC) interface (http://jsoc.stanford.edu/ajax/lookdata.html) at a cadence of 720 seconds, centered at the latitude and longitude of the NOAA AR as specified in the SRS (tracked according to the Carrington rate, the synodic rotation rate of the Sun as observed from Earth), and with a spatial extent of 600 × 600 pixels. This image size was chosen to correspond to approximately 300 arcseconds × 300 arcseconds (300″ × 300″) commensurate with previous work on solar flare prediction, e.g.^37,41,42^, and to be large enough to encompass the typical range of AR sizes^43^. We chose to extract AR images with a consistent image size as that is particularly important for common deep learning architectures for image classification (such as CNNs) which assume a fixed input size (as also discussed in^13,14,21–24,37,38^). The common approach of resizing arbitrarily-sized AR patches (as originally advocated by^21^ and subsequently adopted by^13,14,22,23,25^) can confound AR size (which is an important factor in flare productivity^23,24^) and distort the aspect ratio of ARs, making regions appear more or less sheared than in the original data. The common approach of cropping could remove important information from the AR. The common approach of padding as used in^24,38^ can introduce artifacts and will still confound AR size when resizing the padded square image to a consistent size. We have not explicitly considered the effect of close proximity ARs. Manual inspection of the data reveals ~25% of the dataset contains overlapping AR regions, commensurate with the analysis in^9,11^ which showed 20% of SHARPs^5^ contain contributions from more than one NOAA AR. These images with overlapping content can introduce errors in the machine learning algorithms if they cue on portions of an overlapping AR but are assessed according to the flaring behavior of only the central AR. Any partitioning of the images to mitigate the overlap between AR images, however, would result in inconsistent image sizes. Future work may consider exclusion of images with overlapping AR content, but we do not consider that preprocessing here, similar to^9^. Third, we used the SWPC Event Reports (ER) (ftp://ftp.swpc.noaa.gov/pub/warehouse/) to extract the AR number, peak flare time, and flare strength in order to provide labels for those researchers investigating a supervised classification or regression problem. Figure 1 summarizes the data flow used to create this dataset.

In total, we downloaded images corresponding to 1,655 NOAA ARs which appeared with sunspot structure on the Sun from 01 May 2010 through 31 December 2018, a total of 1,372,004 HMI images from NOAA ARs 11064 through 12731. We only include those ARs which appeared for the totality of their lifetime within the time range 01 May 2010 through 31 December 2018; thus ARs which were already present on the Sun prior to 01 May 2010 or continued their presence on the Sun after 31 December 2018 are not included in this dataset. NOAA ARs 11160, 11171, 12623, and 12705 never developed sunspots and thus contribute no images to this dataset. Additionally, NOAA ARs 11190, 11493, 11494, 11496, 11501, 11503, 12472, 12473, and 12570 are not included in this dataset since they appeared during times when the SDO satellite was missing fine guidance (http://jsoc.stanford.edu/data/cov.html) and thus the location of the ARs could not be accurately tracked. (More specifically, within the JSOC code, a reference time (http://jsoc.stanford.edu/doxygen_html/im__patch_8c-source.html) is specified for the AR corresponding to the time that AR will be at disk center (http://jsoc.stanford.edu/doxygen_html/libs_2astro_2heliographic__coords_8c-source.html) and no data records are returned if there are no valid data within a four hour window of that reference time.) The entire image set (i.e., the 1,372,004 .fits images) comprises 537 GB. We also provide a preconfigured AR dataset of .fits images and corresponding flare labels, which comprises 375 GB and a reduced size (spatially and bit-depth) dataset of .png images and corresponding flare labels, which comprises 15 GB. The preconfigured full-resolution dataset, described below, includes images within ±60° latitude and longitude (to minimize projection effects in the magnetograms) and that contain no NaN pixels, labeled according to flaring behavior within 24 hours and at a flare strength greater than C1.0. The preconfigured reduced resolution dataset, also described below, contains the same images as the preconfigured full-resolution dataset, reduced to 224 × 224 pixels and 8-bit intensities, and the same labels.

### The entire image set

Here we describe the process by which we downloaded the entire image set and corresponding labels. This involved three steps: 1) parsing the SRS for ARs to direct the download process, 2) downloading the magnetogram images, and 3) parsing the ER for flares associated with ARs to provide labels for the images.

#### Parsing the solar region summaries for active regions

We used the NOAA SWPC SRS (ftp://ftp.swpc.noaa.gov/pub/warehouse/) to determine the dates a NOAA AR is visible on disk to direct the download process. The SRS are downloaded as one .txt file per day. We used Part I data in the SRS which detail those active regions with associated sunspot structures (ftp://ftp.swpc.noaa.gov/pub/forecasts/SRS/README). For each NOAA AR appearing in SRS Part I, we store the NOAA AR number, the date the AR first appears in the SRS, and accumulate the total number of days the same AR appears in the SRS. We store these data in a comma separated text file ARList.txt where each line is of the format NNNN,YYYYMMDD,X, where NNNN is the four digit NOAA AR number, YYYYMMDD is the initial date of appearance, and X is an integer number of days. The ARList.txt file used to download the image set described here is provided as part of the GitHub repository at^44^.

#### Downloading the magnetograms for active regions

The text file ARList.txt as described above is used to specify an appropriate date range to download the HMI magnetograms (JSOC data product HMI.M_720s) centered on a given AR. No additional preprocessing, e.g., equal-area projection or correction for projection effects are applied, although such processing could be applied subsequently. Our desire in creating this dataset is to provide minimally proccessed images compatible with machine and deep learning studies. We request HMI magnetograms beginning at time 00:00:00 on the first day the AR appeared through 00:00:00 on the first day the AR disappeared. While there are modules to access SDO data for python (e.g., SunPy^45^) and IDL (e.g., SolarSoft, http://www.lmsal.com/solarsoft/sswdoc/index_menu.html) without navigating the JSOC webpage, the ability to extract and track a cutout around a NOAA AR does not appear to be accessible through any means other than the website. A postprocessing of full-disk images would require significantly more storage space and data transfer; use of the JSOC web interface minimized the data transfer required. In order to automate the process to download the 1,655 ARs, we wrote a python script to interact with the JSOC webpage using the selenium package (https://pypi.org/project/selenium/) and geckodriver (https://github.com/mozilla/geckodriver) for Mozilla’s firefox web browser. We provide this code as part of the GitHub repository^44^, but note that the code will break if any of the underlying html code on the JSOC website changes.

Since the JSOC driver code is fragile, we describe in detail the process of interacting with the JSOC Data Export webpage to download a single AR of data here. Readers who are interested in using the curated datasets^46–48^ described in this paper can skip to the next subsection. Readers who are interested in downloading a custom dataset from the JSOC Data Export webpage may be interested in the process described here. This process assumes that the SWPC SRS have been parsed as in the previous section to determine the beginning date and number of days the AR is on disk.

- Navigate to the JSOC Data Export tool (http://jsoc.stanford.edu/ajax/exportdata.html)
- In the RecordSet field, enter the data locator in the form hmi.M_720s[date1_time1_TAI-date2_time2_TAI][?quality>=0?] where dates and times are in the format YYYY.MM.DD_HH:MM:SS, TAI is the designation for international atomic time used by SDO, and the quality keyword specifies a search only for observables that were created. Press enter and the Record Count field will change to the total number of images spanned by the requested time period. There should be approximately 120 images per day requested.
- Using the Method dropdown menu, select url-tar.
- Check the Enable Processing checkbox which will result in the appearance of several additional check boxes.
- Check the im_patch checkbox which will result in the appearance of an Image Patch Extract box.
- In the Image Patch Extract box:
  - Ensure Tracking is checked in the options row.
  - Specify the NOAA AR number in the options row as a four or five digit number. Press enter and the T_REF, X, and Y fields will populate with reference time and location information for the AR. If the four digit truncated NOAA AR number is entered, the field automatically changes to the corresponding five digit number.
  - Verify T_START and T_STOP match the dates given in the RecordSet field.
  - Verify Cadence matches the cadence specified in the RecordSet field.
  - Verify BoxUnits is set to pixels.
  - Set Width and Height to 600 each.
  - Click the Check Params button which will change the adjacent text field from Not Ready to OK to submit.
- Verify Protocol is set to FITS.
- Enter the user’s email (to which notification will be sent when the data is ready to be downloaded) in the Notify field and the user’s name in the Requestor field. The user’s email must match a registered user (see also next bullet).
- Click Check params for export and the Not Ready To Submit button will change to a Submit Export Request button. If the email entered in the Notify field is not registered, a message will appear specifying that the user should respond to an email from JSOC within 15 minutes to register their email. An email will be sent from jsoc@sun.Stanford.EDU with subject “CONFIRM EXPORT ADDRESS” with further instructions. In short, a simple response to that email will register the user after which the user should receive a second email with subject “EXPORT ADDRESS REGISTERED.” After this initial registration process, the user will need to click the Check params for export button again. This registration process will need to be completed only once per user.
- Click Submit Export Request at which point the RequestID field will be populated with a string used to identify the data request. There may be few second delay before the RequestID field will populate.
- At the bottom of the page in the JSOC Data Export Status and Retrieval section, verify RequestID matches the above given RequestID.
- Periodically click Submit Status Request until the Status field becomes Data Ready. The Status may say Bad Request Status for the first few clicks of Submit Status Request; continue to click the same button until a request time is displayed in the Status field.
- When the Status field becomes Data Ready, click on the link provided in the TarFile Location field to download the requested data.

#### Parsing the event reports for active regions

Using the SWPC Event Reports (ER) (ftp://ftp.swpc.noaa.gov/pub/warehouse/) we parsed the text data for XRA events in the Type column (corresponding to x-ray events detected by the Geospatial Operational Environmental Satellite (GOES) spacecraft) with an associated number in the REG# column (corresponding to a NOAA AR number, see ftp://ftp.swpc.noaa.gov/pub/indices/events/README). This provides the means to associate GOES x-ray flares with NOAA AR numbers. For those x-ray events associated with a NOAA AR, we additionally parsed the ER for the peak flare time (Max column) and flare strength (Particulars column). We store these data in a comma separated text file EventList.txt where each line is of the format YYYY MM DD,HHMM,N NNN,KX.X where YYYY MM DD is the date, HHMM is the time, NNNN is the four-digit NOAA AR number, and KX.X is the GOES strength (e.g., C1.0 or X10.1, see https://svs.gsfc.nasa.gov/10109). The EventList.txt file for this dataset is provided as part of the image set at^48^. The focus of this dataset is on x-ray flares observed by the GOES satellites associated with an AR, but we note that the SWPC ER contain flares observed by other instruments (e.g., optical flares observed in H-alpha) and may contain x-ray flares without an associated AR or with erroneous ARs^7,14,22,49,50^. While this indicates a possibility for this dataset to neglect some flares that are observed (either by other instruments or due to GOES not attributing that flare to an AR), this is consistent with many studies of flare prediction, e.g.^6,8–14,16,18–21,23,25,31,34,37^. We further note that a recent report from June 2022 regarding operational data from the GOES satellites (https://ngdc.noaa.gov/stp/satellite/goes/doc/GOES_XRS_readme.pdf) specifies that flux measurements (and thus flare strengths) on GOES satellites 1–15 should be calibrated by a multiplicative factor of 1/0.7 to match the accurate flux readings of GOES satellites 16+. We have not implemented this correction in these datasets to keep the validation more directly comparable to previous work which uses the directly reported GOES flare strengths. All flare strengths in these datasets are reported from GOES satellites 13–15. This implies that the absolute flare strengths are consistently lower by a multiplicative factor of 0.7 as compared to GOES 16+ flare strengths but that the technical validation herein will not be affected by this consistent linear scaling. Flare sizes in these datasets can be adjusted by a multiplicative factor of 1/0.7 and it is recommended to implement this scaling factor if integrating these data with more recent GOES data to ensure proper calibration. The preconfigured datasets with arbitrary flare strength cutoff of ≥C1.0 for a binary classification will exclude some larger B-class flares that, with the scale factor correction, would meet the ≥C1.0 threshold. As such, validation using these datasets in a binary classification should carefully consider the interpretation of the flare-strength cutoff in light of whether the calibration factor was implemented. As is further discussed below, we define a separate list associating flares to AR images so that the same image dataset can be used for different definitions of flaring behavior, either according to different thresholds of GOES classes, or according to other flaring behavior or catalogs, e.g., those in^50^.

### Preconfigured datasets (full and reduced resolution)

In this section we provide details on the postprocessing of the dataset according to AR location and flaring behavior. We provide a preconfigured dataset consisting of AR magnetograms within ±60° latitude and longitude, containing zero NaN pixels, and labeled according to flaring behavior within 24 hours and at a flare strength greater than C1.0. Additionally, the code available at^44^ can configure a dataset according to different latitude/longitude, acceptable number of NaN pixels, and flaring behavior. As described above, we download magnetogram images for NOAA ARs for the duration of their appearance on the solar disk; hereafter, we refer to this as the “image set” to distinguish it from the “AR dataset.” The preconfigured AR dataset (described below) is available at^46^ and a reduced resolution preconfigured AR dataset (described below) is available at^47^. The image set can be acquired by combining the preconfigured AR dataset^46^ and the extra images dataset^48^ which contains those images removed in the preconfiguration process.

#### Filtering data by latitude, longitude, and not-a-number (NaN) pixels

**Fig. 2:**
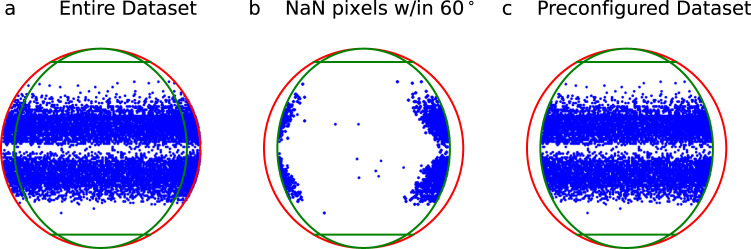
Latitude and longitude of AR centers (blue dots). The red circle denotes the solar radius and the green lines denote ±60° latitude and longitude. (**a**) Latitude and longitude of files for entire dataset (image set). (**b**) Latitude and longitude of files within ±60° and ≥1 NaN pixels. (**c**) Latitude and longitude of files for the preconfigured AR dataset (excluding files outside of ±60° and any files within ±60° with ≥1 NaN pixels). Due to the density of the points and the fact that latitude and longitude are reported at a daily cadence (i.e., multiple images will be reported at the same latitude/longitude), it is not easy to appreciate in panel c the lack of points illustrated in panel b. This is indicative of the fact that the preconfigured dataset contains a wide range of latitudes and longitudes up to ±60°.

**Fig. 3:**
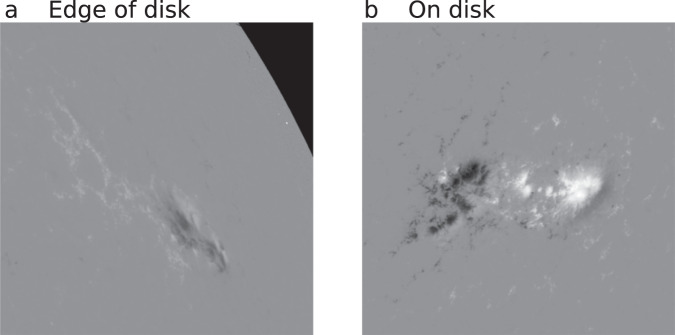
Examples of 600 × 600 pixel magnetogram images, including a disk-edge magnetogram and an on-disk magnetogram. (**a**) Disk-edge magnetogram. NOAA AR 1169, 2011 March 15, 12:00:00. (**b**) On-disk magnetogram. NOAA AR 2396, 2015 August 11, 00:00:00.

Figure 2a shows a scatter plot of the latitude and longitude of the AR centers for the image set. Some of these images, however, are near the edge of the solar disk and parts of the image capture data from off the solar disk (see Fig. 3a). These disk-edge images contain nonsensical magnetic measurements or NaN values. Furthermore, since the HMI magnetograms are LOS, edge-of-disk images are affected by larger projection effects. These projection effects depend not only on the viewing angle but also on the specific geometry of the magnetic field, with deviations from radial in regions of stronger magnetic field introducing larger projection errors^51^. In this dataset, we do not implement any correction for projection effects, e.g., those in^51^, but do provide a means for the user to configure a dataset by restricting the resulting images to reside within latitude and longitude bounds to limit the errors introduced by projection effects. We further note that the user could apply additional preprocessing methods to any of the image set images.

We use the SRS to determine the latitude and longitude for an AR on a given date, noting that the latitude and longitude are provided in the SRS at a daily cadence. Thus, we may exclude some images near the east limb that are just outside of the longitude threshold and rotate into a valid range throughout the day. Similarly, we may include some images near the west limb that are just inside the longitude threshold and rotate out of the valid range throughout the day. Using the daily latitude and longitude provided in the SRS files, we include in the preconfigured AR dataset all images with an AR center within ±60° latitude and longitude (similar to those data in^31,41,42^). We further note that the exclusion of ARs with a latitude or longitude outside of ±60° is primarily motivated by the desire to mitigate projection effects, but also that active regions do not generally extend beyond latitudes of ±60° according to Spörer’s law. A total of 313,601 files, comprising 22.9% of the entire dataset, are excluded from the preconfigured AR dataset based on latitude and longitude; a total of 85 ARs are excluded entirely based on these criteria.

Due to the constant 600 × 600 pixel window of the images, ARs further from the equator may still contain off-disk data and we additionally exclude any image containing any NaN values, an additional 108,356 files and 7.9% of the entire dataset. The majority of these images with NaN values contain a small portion of the disk edge, but there are some images with spurious NaN values from various latitudes and longitudes. Figure 2b shows a scatter plot of those ARs within ±60° latitude and longitude which contained at least one NaN pixel. We note that the majority of these images are near the disk edge, with a higher number of these images clustered near the west limb as compared to the east limb. This is consistent with the expectation that active regions on the west limb will be rotating closer to the disk edge throughout the day and will thus begin to include off-disk NaN data throughout the day.

In total, between the latitude/longitude filtering and the NaN filtering, we exclude 421,957 images, comprising 30.8% of the entire dataset, from the preconfigured dataset. This results in a preconfigured dataset consisting of 950,047 on-disk HMI images (see Fig. 3b) within a range of latitudes and longitudes (see Fig. 2c) from 1,570 ARs. We provide the 950,047 images as part of the preconfigured AR dataset^46^ and the reduced resolution dataset^47^.

#### Assigning flare labels to images

In order to use the dataset for supervised classification or regression, each image in the AR dataset needs a corresponding label specifying whether that image is associated with a flare. We provide a label indicating the flare strength (as a string of GOES strength, e.g., 'C1.0') for images associated with flares or '0' for images associated with non-flaring behavior, using a flare prediction window of 24 hours, and the peak flare time as the time of flare. The user can configure the minimum flare strength as well as the temporal flare prediction window using the dataset customization code available at^44^; any images within the prediction window leading up to a flare are associated with that flare. For those ARs that flare multiple times within the flare prediction window, images are assigned a strength associated with the largest flare, consistent with^7,10,11,15,22,25,32,42^.

**Fig. 4:**
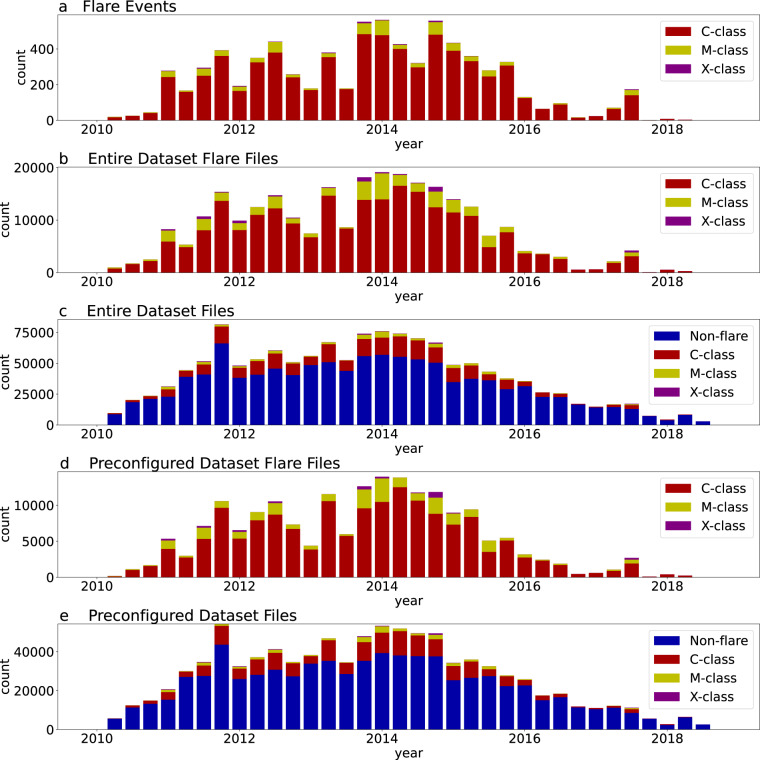
Count of events or files for different flaring behavior versus annual quarter; all flare file counts assume a prediction window of 24 hours. (**a**) Count of flare events in the entire dataset (image set). (**b**) Flare file count for the entire dataset (image set). (**c**) Flare and non-flare file count for the entire dataset (image set). (**d**) Flare file count for the preconfigured dataset. (**e**) Flare and non-flare file count for the preconfigured dataset.

Figure 4a shows a plot of the number of C-, M-, and X-class flares during the timespan of this dataset, while Fig. 4b,c show counts of images associated with flaring behavior for a 24 hour flare prediction window for the entire dataset. We notice very similar trends in the count of flare events (Fig. 4a) and the count of files associated with a flare (Fig. 4b). This indicates that the entire dataset has well-sampled the flaring behavior of the Sun over this time period. In particular, the dataset includes images from across the solar cycle and the trends of flaring behavior have not changed drastically in the preconfiguration process.

In order to assign labels to the AR dataset images, we loop over each event in EventList.txt and assign a label of the GOES strength for all images of the AR within 24 hours of the peak flare time for any flare strengths that satisfy the user-specified minimum flare strength. After assigning flaring images for all events in EventList.txt, all remaining images are labeled '0' to denote non-flaring images. The flare labels are stored in a file KX.X_Hhr_Labels.txt file where KX.X is the user-specified minimum flare strength, e.g., C1.0, and H is the user-specified prediction window in hours, e.g., 24. Each line in the flare labels file is of the form filename,label where filename is the base filename and label is the label (flare strength for flaring and '0' for non-flaring). By maintaining this separate list associating flares to AR images, the same image dataset can be used for different definitions of flaring behavior, either according to different thresholds of GOES classes, or according to other flaring behavior or catalogs, e.g., those in^50^, or to calibrate the flare strengths as outlined in (https://ngdc.noaa.gov/stp/satellite/goes/doc/GOES_XRS_readme.pdf).

For the preconfigured AR dataset, we specify a 24 hour prediction window and a minimum flare strength of C1.0. We provide the C1.0_24hr_Labels.txt file as part of the preconfigured AR dataset^46^ and the C1.0_24hr_png_Labels.txt file as part of the preconfigured reduced resolution AR dataset^47^, both of which contain 190,582 flaring images and 759,465 non-flaring images (950,047 total images). Figure 4d,e show plots of images associated with flaring behavior for the preconfigured AR dataset. We notice very similar trends in the count of flare events for the entire dataset (Fig. 4b,c) and in the preconfigured AR dataset (Fig. 4d,e). This indicates that the configuration of the preconfigured AR dataset based on latitude, longitude, and presence of NaNs in the images has not significantly altered the distribution of flare classes.

#### Full and reduced resolution datasets

The combination of the 950,047 preconfigured images and the labels file constitutes the labeled full resolution dataset; additional helper files are included as described below in Data Records. In addition to the preconfigured full resolution dataset, we have created a reduced resolution dataset at a spatial resolution of 224 × 224 pixels and bit depth of 8 bits capable of representation as an unsigned 8-bit integer (uint8). This reduced resolution dataset contains images in .png format which are more readily ingested by standard image processing libraries and at bit-depth and spatial resolution compatible with typical CNN architectures. Additionally, this dataset requires significantly smaller disk space, making it easier to download.

The spatial resolution is reduced using a standard method of interpolating images to a desired size, the transform.resize command in scikit-image, with parameters order = 1 (bilinear interpolation, the default value), mode = 'reflect' (reflection of intensities at the image edge for interpolation, the default value), clip = True (clip any interpolated values outside of the original data range, the default value), preserve_range = True (keep the range of the original data rather than converting to the convention of intensities in [0, 1]), and anti_aliasing = True (perform lowpass filtering before reducing resolution to avoid aliasing artifacts, the default value). The bit depth of the resized images are reduced by clipping and scaling the intensities to the uint8 range [0, 2^8^−1] = [0,255] and quantizing (rounding) the intensities to the closest integer in [0, 255]. A reduction in bit depth results in error due to both clipping of intensities and the quantization operation. We chose a clipping to the range [−2550, 2550] to affect only 2e-4% of pixels in the entire dataset which originally corresponded to the largest flux values (positive and negative). Then, the scaling operation will result in a range of 20 G being mapped to the same intensity level with an error in the range [−10, 10] G which is on the order of the noise level of the HMI instrument^52^. In order, the intensities are (1) offset by 2550, (2) clipped to [0, 5100], (3) scaled to [0, 255], and (4) rounded to the nearest integer:

1 $${I}_{8}=\left[{\rm{MinMax}}\left(0,5100,I+2550\right)\frac{255}{5100}\right],$$

where *I*_8_ is the image in uint8 bit-depth resolution, *I* is the input image, MinMax(*mn,mx,x*) denotes a clipping of *x* to the range [*mn*,*mx*], and [·] denotes a rounding operation. Note that this is a similar operation to that applied in^24^.

### Dataset partitions

To facilitate comparison between flare prediction methods, we have partitioned the preconfigured datasets into training, validation, and testing sets. To this end we randomly selected 10% of the ARs to set aside for validation purposes (e.g., tuning of algorithm parameters), an additional 10% of the ARs for testing purposes, and the remaining 80% for training purposes. This partitioning by AR alleviates bias associated with random shuffling of images (which can result in the same AR represented in both training and test sets and thus make the “unseen” test dataset appear similar to the training dataset) and chronological splits of the dataset which can lend bias according to different characteristics throughout the solar cycle (as discussed in^18,36^). We note that the initial random assignment of ARs resulted in a validation set with different classification performance, specifically a higher true positive rate (TPR), on several classification tasks. Further investigation found that the validation set had a higher proportion of ARs with very high TPR. Randomly re-assigning seven ARs with TPR > 0.90 from validation to test and a random seven ARs with TPR <0.90 from test to validation resulted in more similar performance between test and validation. The establishment of a standard dataset split for these datasets will facilitate more meaningful comparisons between solar flare prediction methods as all methods can train, validate, and test on the same data. This will render any performance metrics on the test set directly comparable. Future work may consider multiple independent partitions into training, validation, and testing sets as considered in^9,13,15,16,18,22^. There are 157 ARs and 94,757 images in the test data, 157 ARs and 95,933 images in the validation data, and 1,256 ARs and 759,357 images in the training data. Lists of the ARs included in each of the three sets are provided in files List_of_AR_in_Train_Data_by_AR.csv, List_of_AR_in_Validation_Data_by_AR.csv, and List_of_AR_in_Test_Data_by_AR.csv as part of the dataset repositories^46,47^.

## Data Records

The data records for the preconfigured full resolution dataset^46^, preconfigured reduced resolution dataset^47^, and extra images dataset^48^ consist of the following files, also summarized in Table 1. Each dataset contains a directory structure Lat60_Lon60_Nans0, Lat60_Lon60_Nans0_png_224, and active_regions_extra, respectively. This directory structure contains the ARs in four digit directory names, e.g., 1325. Each directory contains multiple magnetogram images in .fits format^46,48^ or .png format^47^. The base filenames are defined with the format hmi.M_720s.YYYYMMDD_HHMMSS_TAI.1.magnetogram as downloaded from JSOC.

**Table 1:** List of files and directory structures in the datasets.

Dataset	Filename/Directory Structure	Description
Preconfigured Full Resolution	Lat60_Lon60_Nans0/	Images (.fits format)
	C1.0_24hr_Labels.txt	Labels for images
	List_of_AR_in_Train_Data_by_AR.csv List_of_AR_in_Validation_Data_by_AR.csv List_of_AR_in_Test_Data_by_AR.csv	Data splits as list of training, validation, and test ARs
	Lat60_Lon60_Nans0_C1.0_24hr_features.csv	Features from images
	Train_Data_by_AR.csv Validation_Data_by_AR.csv Test_Data_by_AR.csv	List of training, validation, and test files for TensorFlow dataloader
Preconfigured Reduced Resolution	Lat60_Lon60_Nans0_png_224/	Images (.png format)
	C1.0_24hr_224_png_Labels.txt	Labels for images
	List_of_AR_in_Train_Data_by_AR.csv List_of_AR_in_Validation_Data_by_AR.csv List_of_AR_in_Test_Data_by_AR.csv	Data splits as list of training, validation, and test ARs
	Lat60_Lon60_Nans0_C1.0_24hr_png_224_features.csv	Features from images
	Train_Data_by_AR_png_224.csv Validation_Data_by_AR_png_224.csv Test_Data_by_AR_png_224.csv	List of training, validation, and test files for TensorFlow dataloader
Extra Images	active_regions_extra/	Images (.fits format)
	EventList.txt	List of flares

The preconfigured dataset^46^ and the reduced resolution dataset^47^ additionally contain the following files of use for classification and regression tasks. In the following, the first filename corresponds to the preconfigured dataset^46^ and the second filename corresponds to the reduced resolution dataset^47^; if only one filename is given, the filenames (and files) are identical between the two datasets.

- C1.0_24hr_Labels.txt, C1.0_24hr_224_png_Labels.txt: a file containing the labels for each of the images in the dataset. The labels are formatted to provide both the regression and classification labels in a form that can be parsed for other applications. Each line in the file is of the form filename,label where filename is the base filename in the image set and label is the label. The label is formatted as a string KX.X for flaring regions, where K is the GOES class (C, M, or X) and X.X is the strength, e.g., 4.7. Non-flaring regions are assigned a label of '0' All labels are assigned for a 24-hour predictive window.
- List_of_AR_in_Train_Data_by_AR.csv, List_of_AR_in_Validation_Data_by_AR.csv, and List_of_AR_in_Test_Data_by_AR.csv: files containing lists of NOAA ARs assigned to the training, validation, and test sets, respectively. Each line in the files is of the format NNNN, the four digit NOAA AR number. Note–these lists are identical between the reduced resolution dataset and the full resolution dataset.
- Lat60_Lon60_Nans0_C1.0_24hr_features.csv, Lat60_Lon60_Nans0_C1.0_24hr_png_224_features.csv: a file with 29 magnetic complexity features extracted from each of the images in the preconfigured datasets. Each line of the file contains 32 comma separated values. The first 29 values are the 29 magnetic complexity features as described below and summarized in Table 2. The last three values are the classification label (1 or 0), regression label (flare strength as as string KX.X or 0), and the base filename. The regression label is formatted as a string KX.X for flaring regions, where K is the GOES class (C, M, or X) and X.X is the strength, e.g., 4.7. All labels are assigned for a 24-hour predictive window.

**Table 2:** List of magnetic complexity feature extracted from the dataset images.

Gradient features	Neutral line features	Wavelet features	Flux features
Gradient mean	NL length	Wavelet energy level 1	Total unsigned flux
Gradient std	NL no. fragments	Wavelet energy level 2	Total signed flux
Gradient median	NL gradient-weighted length	Wavelet energy level 3	Total negative flux
Gradient min	NL curvature mean	Wavelet energy level 4	Total positive flux
Gradient max	NL curvature std	Wavelet energy level 5	
Gradient skewness	NL curvature median		
Gradient kurtosis	NL curvature min		
	NL curvature max		
	NL bending energy mean		
	NL bending energy std		
	NL bending energy median		
	NL bending energy min		
	NL bending energy max
- (Train_Data_by_AR.csv, Train_Data_by_AR_png_224.csv), (Validation_Data_by_AR.csv, Validation_Data_by_AR_png_224.csv), (Test_Data_by_AR.csv, Test_Data_by_AR_png_224.csv): files with labels for each of the images in the preconfigured dataset formatted to provide classification labels in the format expected by a dataframe loader in TensorFlow for the training, validation, and test sets, respectively. Each line is of the form NNNN/filename,label where NNNN is the AR directory, filename is the base filename, and label is the classification label (1 for flaring and 0 for nonflaring). All labels are assigned for a 24-hour predictive window.

The extra images dataset^48^ contains a file EventList.txt which contains the list of events (flares) occurring within the timespan of the dataset. Each line is of the format YYYY MM DD,HHMM,NNNN,KX.X where YYYY MM DD is the date, HHMM is the time, NNNN is the four-digit NOAA AR number, and KX.X is the GOES strength (e.g., C1.0 or X10.1).

## Technical Validation

In this section we describe two experiments that demonstrate the utility of the preconfigured AR dataset. In the first, we implement a flare prediction method using magnetic complexity features and a support vector machine (SVM) classifier. In the second, we provide preliminary results of a transfer learning approach for use of CNNs for flare prediction. The study of temporal evolution of ARs or magnetic features can use this dataset by considering sequences of magnetic features as in^8–12,14,15^ or sequences of images as in^13,25^.

### Magnetic complexity features for machine learning

**Fig. 5:**
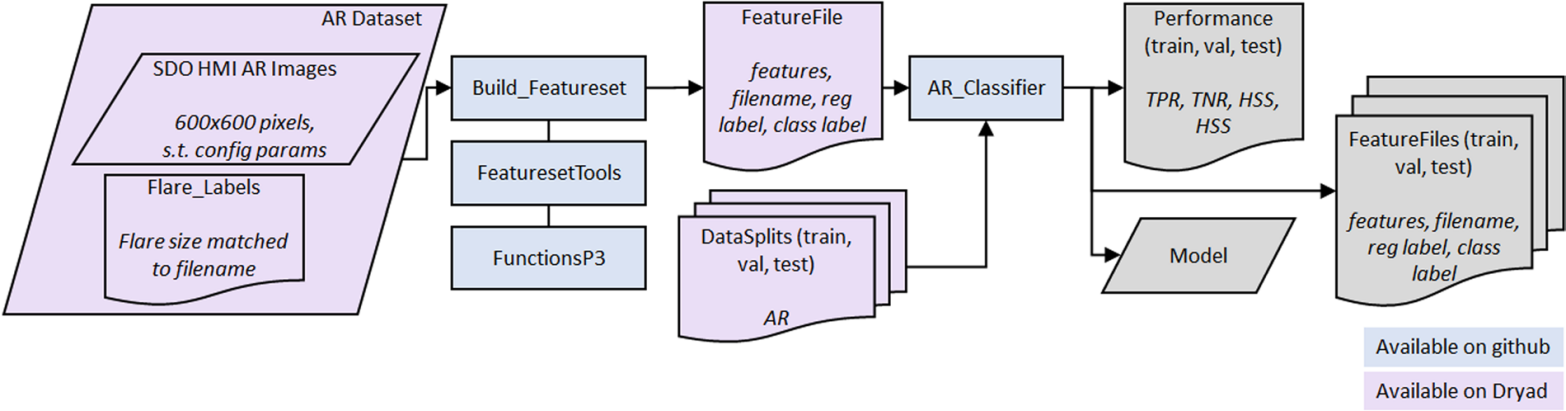
Flowchart of SVM classification of flare activity.

We extract 29 of the 38 magnetic complexity features of^41^ from each of the HMI magnetograms in the preconfigured AR dataset, summarized in Table 2. The 29 magnetic complexity features include 7 gradient features characterizing the spatial gradient of the magnetic flux, 13 neutral line features characterizing the line separating positive and negative flux in the AR, 5 wavelet features characterizing the structure of the magnetic flux at different size scales, and 4 flux features characterizing the total flux in the AR^41^. These features are used as input to an SVM to predict whether the AR will flare within the next 24 hours. An overview of the SVM classification is shown in Fig. 5. The methods presented in^41^ were applied to MDI magnetograms which have lower spatial resolution (~2″ × 2″ pixels), and lower cadence (96 minutes) than the HMI dataset presented here (~0.5″ × 0.5″ pixels and 12 minute cadence). Due the lower cadence of the MDI magnetograms, the dataset was also much smaller, with approximately 260,000 total images (spanning ARs 8809–0933 and 01 January 2000 through 31 December 2006). Nine flux evolution features from^41^ are omitted in this work: these features require a comparison between two images and therefore cannot be directly linked to a single image, the cadence of the HMI magnetograms is 12 minutes (as opposed to 96 minutes) leading to minimal evolution of an AR between images in this dataset, and the flux evolution features proved to be poor features for classifying ARs.

We provide the 29 magnetic features (see also Table 2) as part of the preconfigured AR dataset^46^ and for the reduced resolution dataset^47^ and the code to extract the magnetic features on GitHub at^44^. Each row in the .csv file represents an image in the dataset. The first 29 columns are the 29 magnetic features. The 30th column is the binary flare class ('1' or '0') and the 31st column is the flare strength in terms of the GOES strength (with a value of '0' representing no flare or a flare smaller than 'C1.0'). The last column is the filename of the image corresponding to the magnetic features and flare class.

An SVM classifier is trained on the training set using the SVC function from scikit-learn; this code is also available on GitHub at^44^. All parameters were left as the default (C = 1.0, shrinking = True, probability = False, tol = 0.001, decision_function_shape = 'ovr', break_ties = False, random_state = None) with the exception of the kernel parameter which was set to 'linear' and the class_weight parameter which was set to 'balanced' to account for the imbalanced nature of this dataset. This experiment is intended as a validation of the use of the datasets for classical machine learning methods. As such, we have not optimized the kernel or parameters of the classifier. Performance metrics are evaluated on the test set and are summarized in Table 3. The performance metrics considered are all derived from the four confusion matrix entries encompassing a count of True Positives (TP), True Negatives (TN), False Positives (FP), and False Negatives (FN):

2 $$TPR=\frac{TP}{TP+FN},$$

3 $$TNR=\frac{TN}{TN+FP},$$

4 $$HSS=2\frac{(TP\cdot TN)-(FN\cdot FP)}{(TP+FN)(FN+TN)+(TP+FP)(FP+TN)},$$

5 $$TSS=TPR-(1-TNR).$$

**Table 3:** SVM performance on the test dataset for the full resolution and reduced resolution datasets.

Method	Dataset	TPR	TNR	HSS	TSS	TP	TN	FP	FN
SVM	Full Resolution	0.7484	0.7791	0.4485	0.5275	16,123	57,042	16,173	5,419
	Reduced Resolution	0.7884	0.7464	0.4350	0.5348	16,984	54,650	18,565	4,558

As a comparison the work in^41^ achieved a TPR of 0.81, TNR 0.70, HSS 0.39, and TSS 0.51. Given that work was applied to a different dataset from a different instrument, we find the results here comparable to that work and a validation of the utility of this dataset for flare prediction. We also note that the comparable performance between the full and reduced resolution data indicates that the reduced resolution dataset has retained the vast majority of the information needed for this classification problem. We note, however, that other machine learning tasks may benefit from the increased spatial or bit depth resolution of the full resolution dataset.

### Deep learning

**Fig. 6:**
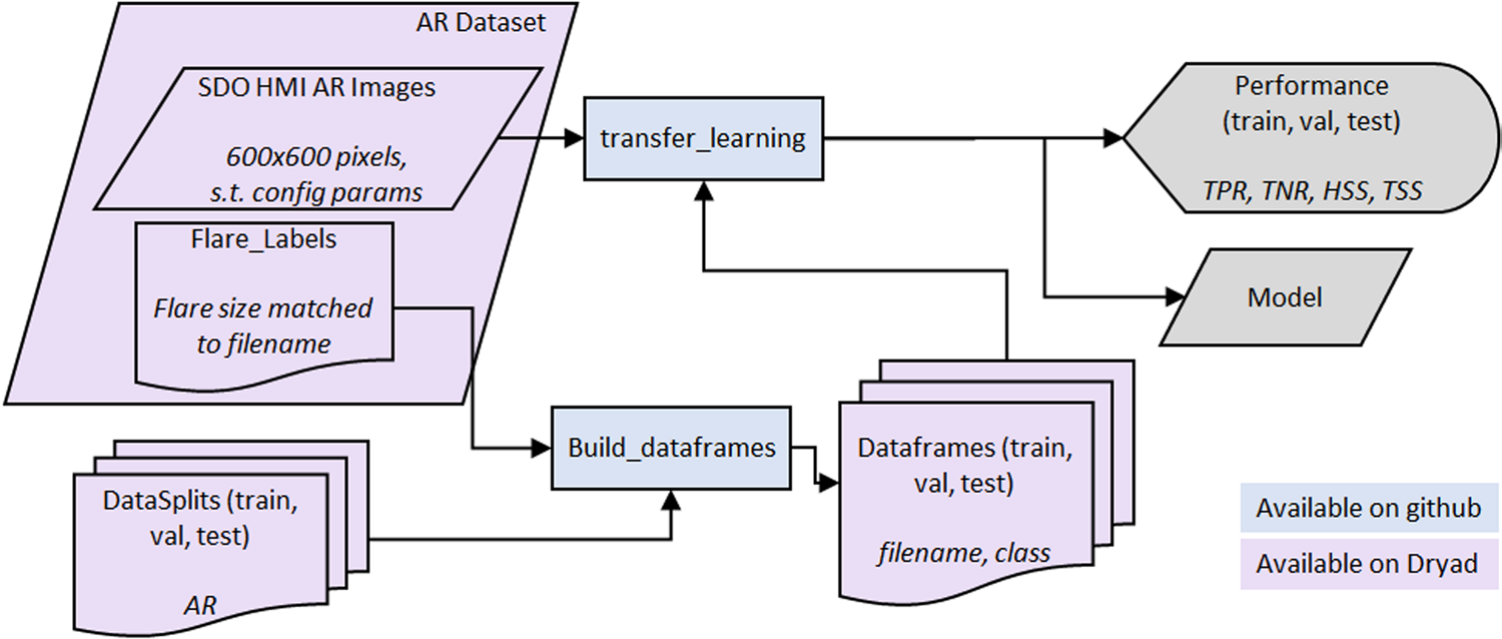
Flowchart of VGG classification of flare activity.

We perform supervised training via transfer learning on the VGG16 CNN^27^, pretrained on ImageNet using the tensorflow.keras (https://www.tensorflow.org) VGG model. An overview of the VGG classification is shown in Fig. 6 and code is available on GitHub at^44^. We replace the final fully connected layer (originally 4096 × 1000) with a 4096 × 2 layer with softmax activation. In training, we freeze all layers except that final fully connected layer. For the full resolution data in .fits format, a custom data generator was written since the .fits format is not one that TensorFlow can handle natively. Within that data generator, the images are resized to the expected spatial dimensions (224 × 224 pixels) using the skimage.transforms.resize command with options order = 1, mode = 'reflect', clip = True, preserve_range = True, and anti_aliasing = True and to the expected intensity range by linearly scaling the full range of the data [−5978.7, 5978.7] to [0, 255]. Note that this intensity rescaling utilizes the full range of intensities without clipping to minimally affect the intensity resolution of the images; this is a different scaling than used in the reduced resolution dataset. The images are then preprocessed with the built-in preprocess_input function as part of the tensorflow.keras VGG model. For the reduced resolution dataset, the flow_from_dataframe method is used along with the VGG preprocess_input preprocessing. Both data generators use a batch size of 64. For training, we used the adam optimizer with options learning_rate = 0.001, beta_1 = 0.9, beta_2 = 0.999, epsilon = 1e-07, and amsgrad = False and the categorical cross-entropy loss. The networks are trained for 5 epochs with the class_weight parameter set to 1 for the majority (non-flare) class and *N*_*n*_/*N*_*f*_ for the minority (flare) class, where *N*_*n*_ is the number of nonflaring examples and *N*_*f*_ is the number of flaring examples. We wrote custom tensorflow.keras metrics to track the TPR, TNR, HSS, and TSS (and the metrics of TP, TN, FP, and FN needed to compute those metrics) throughout the training process. This experiment is intended as a validation of the use of the datasets for deep learning methods. As such, we have not optimized the architecture, which layers are frozen, or optimizer parameters. The best model was chosen as the epoch with the maximum validation TSS. Performance on the test data is summarized in Table 4. We see scores commensurate with the SVM performance, indicating the validity of this dataset in deep learning methods.

**Table 4:** VGG performance on the test dataset for the full resolution and reduced resolution datasets.

Method	Dataset	TPR	TNR	HSS	TSS	TP	TN	FP	FN
VGG	Full Resolution	0.6952	0.8142	0.4567	0.5094	14,977	59,611	13,604	6,565
	Reduced Resolution	0.7344	0.7980	0.4636	0.5325	15,822	58,428	14,787	5,720

## Usage Notes

Further details on usage of the datasets can be found as part of the dataset repository documentation for the preconfigured dataset^46^, reduced resolution dataset^47^ and extra images dataset^48^. Further details on usage of the code for configuration of the datasets and classification can be found as part of the GitHub repository^44^.

## Data Availability

All code used to generate and manipulate the dataset, as well as code used in the Technical Validation is available at the GitHub repository^44^. Further details and documentation regarding code usage are included therein.
